# Obesity Increases Gene Expression of Markers Associated With Immunosenescence in Obese Middle-Aged Individuals

**DOI:** 10.3389/fimmu.2021.806400

**Published:** 2022-01-05

**Authors:** Diego T. Brunelli, Vinicius O. Boldrini, Ivan L. P. Bonfante, Renata G. Duft, Keryma Mateus, Leonardo Costa, Mara P. T. Chacon-Mikahil, Ana M. Teixeira, Alessandro S. Farias, Cláudia R. Cavaglieri

**Affiliations:** ^1^ Exercise Physiology Lab (FISEX) - Faculty of Physical Education, University of Campinas (UNICAMP), Campinas, Brazil; ^2^ Autoimmune Research Lab, Department of Genetics, Microbiology and Immunology, Institute of Biology, University of Campinas (UNICAMP), Campinas, Brazil; ^3^ Research Center for Sports Sciences and Physical Activity, University of Coimbra, Coimbra, Portugal

**Keywords:** type 2 diabetes mellitus, inflammation, aging, physical fitness, adipose tissue

## Abstract

Recently, it has been argued that obesity leads to a chronic pro-inflammatory state that can accelerate immunosenescence, predisposing to the early acquisition of an immune risk profile and health problems related to immunity in adulthood. In this sense, the present study aimed to verify, in circulating leukocytes, the gene expression of markers related to early immunosenescence associated with obesity and its possible relationships with the physical fitness in obese adults with type 2 diabetes or without associated comorbidities. The sample consisted of middle-aged obese individuals (body mass index (BMI) between 30-35 kg/m²) with type 2 diabetes mellitus (OBD; n = 17) or without associated comorbidity (OB; n = 18), and a control group of eutrophic healthy individuals (BMI: 20 - 25 kg/m²) of same ages (E; n = 18). All groups (OBD, OB and E) performed the functional analyses [muscle strength (1RM) and cardiorespiratory fitness (VO_2max_)], anthropometry, body composition (Air Displacement Plethysmograph), blood collections for biochemical (anti-CMV) and molecular (gene expression of leptin, IL-2, IL-4, IL-6, IL-10, TNF-α, PD-1, P16^ink4a^, CCR7, CD28 and CD27) analyses of markers related to immunosenescence. Increased gene expression of leptin, IL-2, IL-4, IL-10, TNF-α, PD-1, P16^ink4a^, CCR7 and CD27 was found for the OBD and OB groups compared to the E group. Moreover, VO_2max_ for the OBD and OB groups was significantly lower compared to E. In conclusion, obesity, regardless of associated disease, induces increased gene expression of markers associated with inflammation and immunosenescence in circulating leukocytes in obese middle-aged individuals compared to a eutrophic group of the same age. Additionally, increased adipose tissue and markers of chronic inflammation and immunosenescence were associated to impairments in the cardiorespiratory capacity of obese middle-aged individuals.

## Introduction

Obesity is considered a global health problem, affecting a large part of the population and becoming one of the main causes of reduced quality of life, morbidity and mortality (1–3). Several studies have proposed that the central mechanism underlying obesity and its related comorbidities comes from a persistent state of low-grade chronic inflammation together with dysregulation in the inflammation-stress feedback mechanisms (4–6), mainly due to increased production of pro-inflammatory markers such as leptin, interleukin-6 (IL-6), tumor necrosis factor -α (TNF-α), C-reactive protein (CRP) and monocyte chemotactic protein - 1 (MCP1) in the withe adipose tissue, together with the M1 macrophage phenotype infiltration in this tissue associated with obesity (6–8).

Furthermore, it has been proposed that dysfunctional adipocytes are involved in the production of oxidative stress through the generation of reactive oxygen species, implicating further inflammation and tissue damage (9). Additionally, there is a consensus that an appropriate defensive immune response requires low levels of oxidation and inflammation interconnected with many feedback loops (10). However, cell damage was seen to appear when these levels were increased (as in the case of obesity) and innate immunity cells, such as macrophages, exhibit an over-activated production of oxidative and inflammatory compounds which occurs mainly in the absence of antigenic stimulus and cannot be neutralized by antioxidant nor anti-inflammatory defenses, thus leading to a chronic state of oxidative and inflammatory stress (5, 10).

In addition, increase evidence suggests that the process of aging is the result of the accumulation of cellular damage caused by oxidative and inflammation stress throughout the lifetime of an organism, where the immune system seems to be involved in this stress and in the rate of aging (5, 11). Thus, given the context of neuro-immune-endocrine communication and its changes in oxidative and inflammatory situations, as well as the impairment of the immune system, characteristics similar to those observed in aging, it has been speculated that obesity generates premature immunosenescence (biological aging of the immune system), and this may be one of the reasons for the increased rate of morbidity and risk of death related to this disease (12–15).

Immunosenescence has a number of hallmark features such as increased activities of immune cells and progressive decline in the functional activity of phagocytic cells, natural killer cells, lymphoproliferative response and mitogen-stimulated cytokine production (16–18), accumulation of late-differentiated subsets of T lymphocyte accompanied by lowered proportions of naïve T cells and seropositivity for latent cytomegalovirus (CMV) and Epstein-Barr virus (EBV) infections (15, 18, 19), these changes being associated with poor vaccine efficacy, decreased immune surveillance and increased morbidity and mortality as a result of infectious diseases (19–21). In fact, studies show that obese individuals have a higher prevalence and severity of persistent viral infections, such as the herpes simplex virus type 1 - (HSV1) (22) and SARS-CoV-2 (21) and a lower immune response to infections by pathogens and vaccines (23, 24), thus indicating the presence of a less competent immune system when compared to non-obese individuals with similar age.

Furthermore, it has been postulated that the presence of senescent cells may be the causative agent in the development and progression of type 2 diabetes (T2D) and contributes to tissue dysfunction; however, the diabetic milieu might permit the development of senescent cells (25). Additionally, it has been shown that increased presence of senescent T cell has a detrimental impact on the immune function of obese people living with T2D (26). Thus, the need for studies that investigate the adverse effects of obesity with or without associated comorbidity on the various factors related to immunosenescence is evident. Given this context, the present study aimed to verify the effects of obesity per se or with associated comorbidity T2D on the gene expression of pro and anti-inflammatory and senescence markers of the immune system in circulating leukocytes and their correlations with physical fitness in obese middle-aged adults with T2D or without associated disease.

## Materials and Methods

### Subjects and Study Design

The project was publicized by means of fliers, posters in the university campus, internet, advertising in city newspapers and by visits to places such as public town squares and malls. The inclusion criteria adopted were as follows: middle-aged (40 – 60 years old) male or female classified as obese [(body mass index (BMI) > 30 kg∙m^-2^)] with T2D or with absence of an associated disease or middle aged individuals of both sexes´ eutrophics (BMI between 20 - 25 kg/m²) who had not participated in regular exercise and/or dietary programs over the last 12 months preceding the start of the study, according to the Baecke Habitual Physical Activity Questionnaire (27). Furthermore, volunteers should be non-smokers. Specifically in relation to the female sex, the volunteers had to be postmenopausal.

Initially, 228 subjects were recruited; however, 145 were excluded at the initial interview for not meeting the inclusion criteria and/or for having any feature covered by the exclusion criteria. Exclusion criteria included the following: coronary artery disease, severe hypertension, type 1 diabetes mellitus or insulin-dependent, chronic obstructive pulmonary disease, limiting osteo-articular diseases, or were using any medication that could interfere in the physiological responses of evaluations. Of the 83 subjects who were selected, 65 were approved on clinical examination and exercise electrocardiogram (ECG); however, 12 volunteers were excluded according to the discontinuity criteria which were: lack of motivation or availability of the volunteer in attending the evaluation sessions or other risks that might occasionally arise, even after clinical release. Thus, after the initial evaluations, the middle-aged volunteers were allocated to each specific group: obese with T2D (OBD; n = 17; 7 males and 10 females); obese with absence of associated disease (OB; n = 18; 9 males and 9 females); and the eutrophic group (E; n = 18; 9 males and 9 females). All volunteers in the OBD group were using metformin to treat T2D.

All groups (OBD, OB and E) performed the functional analyses [muscle strength (1RM) and cardiorespiratory fitness (VO_2max_)], anthropometry, body composition (Air Displacement Plethysmograph), blood collections for biochemical (anti-CMV) and molecular (gene expression of leptin, IL-2, IL-4, IL-6, IL-10, TNF-α, PD-1, P16^ink4a^, CCR7, CD28 and CD27) analyses of markers related to immunosenescence at baseline. All evaluations were performed with the volunteers under conditions of spontaneous breathing of atmospheric air, in a room with an average ambient temperature of 23° C. In order to familiarize themselves with the location, tests and equipment used, the volunteers were invited to visit the premises used for the evaluations and an explanation of the procedures to be used were given.

All the experimental methods and procedures adhered to the Helsinki declaration and were all approved by the local University Research Ethics Committee (CAAE: 55952516.6.0000.5404). Additionally, all participants were informed about the purpose and risks of the study and signed an informed consent document before participation.

### Anthropometric Measures and Body Composition

Height was measured using a wall-mounted stadiometer with a precision of 0.1 cm, and the weight was taken using a calibrated manual scale (Filizola^®^ S.A., São Paulo, SP, Brazil) with a precision of 0.1 kg. The BMI was calculated by dividing body mass (kg) by height squared (m²). Waist circumference was measured at the midpoint between the last ribs and the iliac crests with an inelastic metric tape, with the precision of 0.1 cm. The measurement was performed in triplicate by the same trained professional and the average of these three measurements was calculated.

Body composition of the volunteers was estimated by plethysmography in the Bod Pod ™ (COSMED USA, Inc., Concord, CA) body composition system. The same investigator performed all measurement assessments.

### Dietary Intake Assessments

Food records were given to the participants by trained researches who instructed them individually through a presentation of an already completed model food record and photographs of model home measures. They were instructed to complete food records for three nonconsecutive days (two days in the week and one day at the weekend) and the mean of the three food records was used as the dietary intake of each subject. Total calories, carbohydrates, lipids and protein were calculated using the DietPro^®^ Software program, version 5i (Viçosa, Minas Gerais/Brazil).

### Maximal-Strength Assessments

Maximal-strength for upper- and lower-body was measured by a one-repetition maximum (1RM) test on bench press and leg press. The test protocol was conducted on RIGUETTO^®^ equipment’s (São Paulo/Brazil). Subjects were required to perform 10 repetitions at 50% of their estimated 1RM (according to each participant’s capacity). After 3 min of rest, subjects were required to perform 3 repetitions at 70% of their estimated 1RM. Another 3 min of rest was applied and then subsequent trials were performed for 1RM with progressively heavier weights until the 1RM was determined within three attempts, with 3–5 min of rest between trials (28).

### Cardiorespiratory Assessment

The cardiorespiratory assessment was performed using a progressive effort protocol on a treadmill (Quinton, model TM55, USA), with a continuous collection of expired gases breath to breath (CPX Ultima, MedGraphics, USA). The cardiorespiratory fitness verified by the maximum oxygen consumption (VO_2max_) was determined by the average values of the last 30 s of the test (29).

### Blood Sampling and Analysis

Approximately 10 ml of blood was collected from the antecubital vein into Vacutainer^®^ tubes (Becton Dickinson Ltd, Oxford/England) for plasma samples (containing anticoagulant EDTA), in the morning (07:00 – 09:00 a.m.), after a 12 hour overnight fast and at least 72 hours before the physical and functional evaluations. Additionally, all individuals were instructed not to consume caffeine and alcohol 24 hours before collection. All samples were collected, processed, divided into aliquots, and stored at -80°C for subsequent analysis. Peripheral blood leukocytes were separated by density gradient centrifugation using Histopaque ^®^ (*Histopaque* ^®^ - 1077, Sigma, MO, USA). Plasma glucose analysis was performed using the GOD-Trinder method. Anti-CMV analysis was determined by enzyme-linked immunosorbent assay (ELISA), according to the specifications of the manufacturer (Novus Biologicals, catalog number KA1452, Abnova^®^ Corporation, USA).

Additionally, the extraction of the total leukocyte mRNA was performed following the instructions of the Trizol^®^ reagent. At the end of the extraction of the total RNA, the purity and quantity of the RNA of the samples were verified in the Gene Quant^®^ spectrophotometer (Pharmacia Biotech), paying attention to the 260/280 ratio (possible contamination with protein). All the ratios found in the samples were between 1.8 - 2.0 and the samples were considered optimal for use. For the production of the cDNA, we used the High Capacity cDNA Reverse Transcription Kit^®^ (Applied Biosystems, CA, USA), and followed the manufacturer’s instructions. The gene expression of leptin, IL-2, IL-4, IL-6, IL-10, TNF-α, PD-1, P16ink4a, CCR7, CD28 and CD27 of circulating leukocytes was performed in duplicate using the Real Time Polymerase Chain Reaction (RT-PCR) technique, following the descriptions of Pradella etal. (30). The human HPRT primer was used as the reference gene for the reaction (endogenous control). A minimum of 15 ng cDNA per reaction was used for all genes in order to ensure the efficiency of the RT-PCR reaction. All cDNAs were placed in a set of primers and a probe marked with SYBR Green. As for the list of primers, the following 5´ and 3´ sequences were used: HPRT (forward: GACCAGTCAACAGGGGACAT, reverse: AACACTTCGTGGGGTCCTTTTC); Leptin (forward: GGCTTTGGTCCTATCTGTCTTATGTTC; reverse: CCTGTTGATAGACTGCCAGAGTCTG); CD27 (forward: ACTACTGGGCTCAGGGAAAGCT; reverse: GGATCACACTGAGCAGCCTTTC); CD28 (forward: GAGAAGAGCAATGGAACCATTATC; reverse: TAGCAAGCCAGGACTCCACCAA); P16^INK4a^ (forward: GGGGGCACCAGAGGCAGT; reverse: GGTTGTGGCGGGGGCAGTT); PD–1 (forward: CGTGGCCTATCCACTCCTCA; reverse: ATCCCTTGTCCCAGCCACTC); IL-2 (forward: GAATCCCAAACTCACCAGGATGCTC; reverse: TAGCACTTCCTCCAGAGGTTTGAGT); IL-6 (forward: GGTACATCCTCGACGGCATCT; reverse: GTGCCTCTTTGCTGCTTTCAC); IL-10 (forward: CCGTGGAGCAGGTGAAGAATG; reverse: AGTCGCCACCCTGATGTCTC); IL-4 forward: AACTTTGTCCACGGACACAAGTGC; reverse: GAATCGGATCAGCTGCTTGTGCCT); CCR7 (forward: GCCCAGATGGTTTTTGGGTTC; reverse: GCAAGGTACGGATGATAATGAGG); TNF–α (forward: CGAGTCTGGGCAGGTCTACTTT; reverse: AAGCTGTAGGCCCCAGTGAGTT).

### Statistical Analysis

Initially, the data was organized according to the following groups: Obese diabetic (OBD) or Obese without associated disease (OB) or Eutrophic (E). Then, the data distribution was tested with the Shapiro-Wilk test. From data distribution (parametric or non-parametric), verification of normality and the presence of outliers, the appropriate statistics were applied to verify the differences between the groups studied: one-way Analysis of Variance (ANOVA) for the parametric variables (age, mass, height, waist circumference, VO_2max_, bench press, leg, fat mass, fat-free mass, carbohydrate, protein, lipids and total calories), and when an interaction was observed, Tukey’s *post-hoc* test was applied; Kruskal-Wallis test for non-parametric variables (BMI, blood glucose and gene expression of leptin, IL-2, IL-4, IL-6, IL-10, TNF-α, PD-1, P16^ink4a^, CCR7, CD28 and CD27), and when an interaction was pointed out, Dunn-Bonferroni’s *post-hoc* test was applied. The association between physical fitness and body composition with the gene expression of the immunosenescence markers used in the present study was tested by Spearman’s correlation test. All the data were analyzed using the IBM^®^ SPSS^®^ Statistics 20.0 (IBM Corp. ^©^ Copyright IBM Corporation, USA) software program. The level of significance was set at p≤ 0.05. All data are presented in values of mean ± SD.

## Results

All volunteers did not perform any systematic physical activities in the period prior to the study and were classified as irregularly active according to the Baecke questionnaire, with the times of weekly physical activity reported as follows: OBD = 65.03 ± 8.08 min; OB = 63.13 ± 7.08 min; and E = 68.50 ± 9.30 min. Furthermore, all volunteers (with the exception of 1 individual in the E group) presented serum positivity for CMV. Thus, we do not separate the results between CMV seropositive or negative individuals.

**Table 1** presents the results for anthropometric measurements, body composition and fasting glucose. Significant differences were found for weight, BMI, body fat, fat-free mass, waist circumference and VO_2max_ for OBD and OB as compared to E (p < 0.0001 for all the comparisons). However, there were no differences between OBD and OB for these same variables (p > 0.05). Additionally, as expected, fasting glucose was significantly higher for OBD as compared to OB (p < 0.0001) and E (p < 0.0001), without differences between OB and E (p > 0.05). Furthermore, no significant differences were observed in age, height and 1RM of the bench press and leg press between the groups studied (p > 0.05).

**Table 1:** Anthropometric measurements, body composition, functional capabilities and fasting glucose of obese diabetic (OBD) or Obese without associated disease (OB) or Eutrophic (E) middle-aged individuals.

	OBD (n = 17)	OB (n = 18)	E (n = 18)
Age (years)	52.4 ± 5.0	50.2 ± 5.5	52.5 ± 4.4
Height (m)	1.66 ± 0.08	1.69 ± 0.10	1.69 ± 0.11
Weight (Kg)	88.6 ± 9.3^&^	93.6 ± 13.3^&^	68.2 ± 10.1
BMI (kg/m^2^)	32.3 ± 2.2^&^	32.5 ± 1.5^&^	23.8 ± 1.4
Body fat (%)	39.9 ± 6.4^&^	43.9 ± 7.7^&^	26.8 ± 8.1
Fat-free mass (%)	60.1 ± 6.3^&^	56.1 ± 7.7^&^	73.3 ± 8.0
Waist circumference (cm)	101.6 ± 5.7^&^	101.3 ± 8.2^&^	85.1 ± 8.1
Fasting glucose (mg/dL)	142.1 ± 36.6*^&^	89.3 ± 8.8	87.5 ± 7.6
VO_2max_ (ml/kg/min)	20.92 ± 4.59^&^	22.43 ± 5.33^&^	29.30 ± 4.67
1RM – bench press (Kg)	31,5 ± 14,6	31,4 ± 17,8	25,7 ± 13,6
1RM - leg press (kg)	208,8 ± 57,8	204,7 ± 62,5	165,3 ± 46,5
Diabetes duration (years)	5,2 ± 3,1	–	–

BMI, body mass index. *Significantly different from OB; **^&^** Significantly different from E; p ≤ 0.05.

**Table 2** presents the results for dietary intake assessments. Carbohydrate ingestion was significantly higher for OBD as compared to OB (p = 0.038) and E (p = 0.001). Additionally, protein ingestion was significantly lower for OB as compared to E (p = 0.016). Furthermore, a tendency of higher total caloric ingestion was observed for OBD (p = 0.063) and OB (0.060) in comparison to E. There was no significant difference in lipids ingestion between groups (p > 0.05).

**Table 2:** Carbohydrates, protein, lipids and total calories ingestion of obese diabetic (OBD) or Obese without associated disease (OB) or Eutrophic (E) middle-aged individuals.

	OBD (n = 17)	OB (n = 18)	E (n = 18)
Total calories (Kcal)	1836.4 ± 464.2	2122.0 ± 760.3	1595.8 ± 454.7
Carbohydrates (g)	342.5 ± 91.9*^&^	250.2 ± 112.2	200.8 ± 71.7
Lipids (g)	62.9 ± 21.4	74.3 ± 33.1	58.4 ± 20.8
Protein (g)	80.1 ± 21.5	70.2 ± 15.1^&^	96.1 ± 30.0

*Significantly different from OB; **^&^** Significantly different from E; p ≤ 0.05.

**Figure 1:**
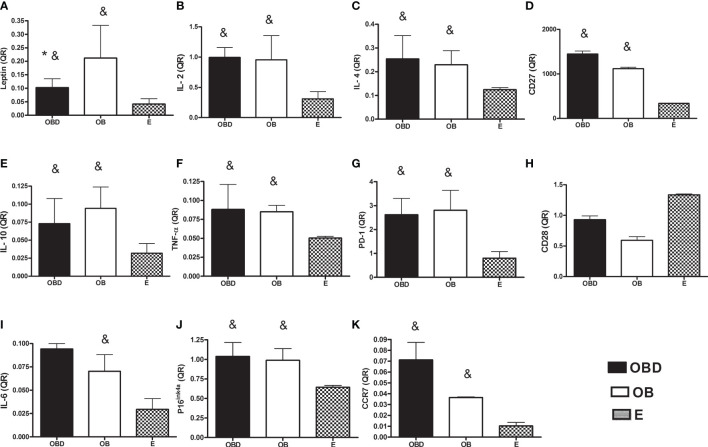
Gene expression of leptin **(A)**, IL-2 **(B)**, IL-4 **(C)**, CD27 **(D)**, IL-10 **(E)**, TNF-α **(F)**, PD-1 **(G)**, CD28 **(H)**, IL-6 **(I)**, P16^ink4a^ **(J)** and CCR7 **(K)** in PBMCs of obese diabetic (OBD) or Obese without associated disease (OB) or Eutrophic (E) middle-aged individuals.* Significantly different from OB; **^&^** Significantly different from E; p ≤ 0.05.

**Figure 1** represents the values of quantifications relative (QR) to endogenous control (HPRT) of the gene expression of the markers analyzed in the present study. Leptin expression was significantly higher for the OBD and OB groups compared to E (p = 0.001 and p = 0.047, respectively; **Figure 1A**) and also for OBD compared to OB (p = 0.020; **Figure 1A**). Additionally, gene expression of IL-2 (p = 0.003 and p = 0.0001, respectively; **Figure 1B**), IL4 (p = 0.001 and p = 0.011, respectively; **Figure 1C**), CD27 (p = 0.014 and p = 0.043, respectively; **Figure 1D**), IL10 (p = 0.001 and p = 0.0001, respectively; **Figure 1E**), TNF-α (p = 0.001 and p = 0.029, respectively; **Figure 1F**), PD-1 (p = 0.006 and p = 0.003, respectively; **Figure 1G**), P16^ink4a^ (p = 0.014 and p = 0.029, respectively; **Figure 1J**) and CCR7 (p = 0.0001 and p = 0.012, respectively; **Figure 1K**) were significantly higher for OBD and OB compared to E, with no difference between OBD and OB for these same variables (p > 0.05 for all the comparisons). On the other hand, gene expression of IL-6 showed significant higher only for the OB group compared to E (p = 0.038; **Figure 1I**), with no difference when comparing the OBD group to OB (p = 1.00) and E (p = 0.253). There were no significant differences for CD28 in the comparison between the groups studied (p> 0.05 for all comparisons; **Figure 1H**).

As regards the correlations between the physical fitness and body composition variables, significant negative correlations were observed between VO_2max_ and BMI (r = -.504, p = 0.0001) and VO_2max_ and body fat (r = -.762, p = 0.0001). In addition, a significant correlation was found between VO_2max_ and fat-free mass (r = .762, p = 0.0001). Furthermore, the correlations between physical fitness and body composition with the gene expression of the immunosenescence markers analyzed in the present study are shown in **Table 3**.

**Table 3:** Correlations between physical fitness and body composition with gene expression of the immunosenescence markers of obese diabetic (OBD) or Obese without associated disease (OB) or Eutrophic (E) middle-aged individuals.

		Leptin	IL-2	IL-4	IL-10	TNF-α	PD-1	IL-6	P16^ink4a^	CCR7	CD28	CD27
BMI	Spearman’s correlation	.498*	.515*	.387*	.483*	.250	.360**	.271	.241	.570*	-.137	.414*
	Sig. (2-tailed)	.000	.000	.005	.000	.086	.010	.055	.088	.000	.353	.002
VO_2max_	Spearman’s correlation	-.373*	-.467*	-.305**	-.357**	-.258	-.321**	-.197	-.169	-.485*	.148	-.517*
	Sig. (2-tailed)	.007	.001	.033	.012	.083	.026	.175	.246	.000	.327	.000
Chest press	Spearman’s correlation	.051	.101	.038	.251	.212	-.012	.077	.255	.125	.187	-.018
	Sig. (2-tailed)	.726	.495	.795	.082	.157	.933	.602	.077	.404	.220	.904
Leg press	Spearman’s correlation	.171	.206	.157	.353**	.191	.074	.112	.170	.258	.063	.067
	Sig. (2-tailed)	.239	.166	.292	.014	.209	.619	.455	.249	.084	.682	.653
Body fat	Spearman’s correlation	.488*	.439*	.339**	.339**	.256	.395*	.194	.164	.369**	-.147	.456*
	Sig. (2-tailed)	.000	.002	.018	.018	.089	.006	.186	.265	.011	.330	.001
Fat free mass	Spearman’s correlation	-.487*	-.437*	-.337**	-.338**	-.252	-.395*	-.197	-.162	-.369**	.144	-.456*
	Sig. (2-tailed)	.000	.002	.019	.019	.095	.006	.180	.272	.011	.341	.001

*Correlation is significant at the 0.01 level (2-tailed); **Correlation is significant at the 0.05 level (2-tailed); n=53.

## Discussion

Over time, scientific evidences have elucidated that obesity is a complex disorder and a major risk factor for many diseases and health problems such as cardiovascular diseases, T2D, cancer, among others (31). Additionally, based on similar characteristics for cellular senescence as those observed in aging, obesity has been linked to the development of early immunosenescence and decreases in life span (12–15, 31). In animal models used in experimentation, Hunsche et al. (5) observed that macrophage and lymphocyte chemotaxis, macrophage phagocytosis, NK cell activity, lymphocyte proliferative response, secretion of IL-1β, TNF-α, IL-6, IL-2 and IL- 10 in leukocyte cultures, as well as the antioxidant and oxidative capacity of obese adult rats were significantly impaired when compared to non-obese adult rats and similar to elderly rats, thus concluding that obesity can generate premature immunosenescence that is aggravated as the obese adult rat ages. In agreement with these finding, studies have shown that obesity and diabetes accelerate biological ageing and consequently the appearance of age-related diseases especially through premature induction of the senescent state (26, 31–33). Results presented herein show that obesity, regardless of associated disease (such as type 2 diabetes mellitus), induces increased gene expression in circulating leukocytes of markers related to chronic inflammation and to senescence of the immune system in obese middle-aged individuals compared to a eutrophic group of the same age. Furthermore, we observed that obesity could lead to impairments in cardiorespiratory fitness which has significant correlations with inflammation and the immunosenescence markers investigated in the present study.

Several studies have shown that the dysfunction in adipose tissue resulting from obesity leads to a chronic inflammatory state firstly in all of the adipose tissue but becoming systemic through the release of pro-inflammatory adipokines and cytokines such as Leptin, TNF-α, IL-6, IL-8, CRP, MCP1, among others in the peripheral blood circulation (6–8, 34). This chronic inflammation associated with obesity directly impacts innate and adaptive immunity cells with both a pro-inflammatory orientation of immune cells in the basal state and results in a decrease in their ability to respond to infection and re-infection (6, 34). In accordance with the previous statements, our results showed increases in gene expression both for markers related to chronic inflammation (leptin, TNF-α, IL-2 and IL-6) and for markers related to senescence of the immune system (PD-1, P16^ink4a^, CCR7 and CD27) in circulating leukocytes of obese middle-aged individuals when compared to lean individuals of equal age and similar physical exercise habits, regardless of having an associated disease. Despite this, we did not find significant differences as regard CD28 expression. However, the eutrophic individuals showed a tendency to express higher levels of CD28, in circulating leukocytes, than obese and diabetic individuals. Lack of CD28 expression has been suggested as an important marker of senescent T cells (35).

We also observed that anti-inflammatory markers (as in the case of IL-10 and IL-4) were more expressed in the circulating leukocytes of obese individuals with or without diabetes when compared to lean individuals. This may be explained, in part, by the overexpression of leptin found in the present study, since the systemic increase in this adipokine increases the proliferation of B lymphocytes, decreases its apoptosis and activates its secretion of cytokines such as TNF-α, IL-6 and IL-10 (36). Additionally, IL-4 is one of the signature cytokines of type II inflammatory response and is the key regulator of activation, growth and differentiation of lymphocyte functions (37). Furthermore, studies have shown that IL-4 and IL-13 mediated IL-4 receptor (IL-4R) signaling in both mouse and human neutrophils inhibits their migration and effector functions *in vitro* and *in vivo* (38–40). Although IL-4 plays a protective role in inflammation by suppressing pro-inflammatory cytokine production like IFN-gamma, TNF-α and IL-1 (41), our results herein may indicate that obesity can contribute to a cascade of pro-inflammatory cytokines that IL-4 are unable to suppress. Thus, according to other studies that also found an increase in IL-4 associated with obesity (42), we speculate that increased expression of IL-4 in the obese groups compared to lean subjects may be due to a compensatory mechanism trying to maintain cell functions, homeostasis and tissue integrity.

In our study, obese middle-aged individuals with T2D or without any associated disease showed higher expression of senescent markers such PD-1 and P16^ink4a^ when compared to lean middle-aged subjects, thus indicating the presence of a premature immunosenescence associated with obesity once PD1 is primarily associated with immune exhaustion (43). In agreement with our results, Wang etal. (44) demonstrated for the first time that obesity increases T cell aging resulting in higher PD-1 expression and dysfunction, which is driven, at least in part, by leptin signaling. In addition, the expression of P16^ink4a^ increases markedly with aging in most mouse tissues and in human skin and kidney tissues (45, 46), which suggests the importance of this tumor suppressor in aging and senescence (47, 48). In addition, overexpression of P16^ink4a^ has been reported in senescent fibroblasts (49), in response to oxidative stress (50, 51), DNA damage and changes in the structure of chromatin (52, 53). However, there is currently no complete understanding of the signs that trigger senescence and, although P16^ink4a^ appears to be one of the main factors in senescence, more information is needed to determine the exact role of each factor in this process (54). Thus, the involvement of PD-1 and P16^ink4a^ during inflammation may contribute to increased cell senescence in obesity and type 2 diabetes (55).

The co-receptors CD28 and CD27 have frequently been used to define sub-populations of memory cells, and historically, down-regulation of CD28 was typically associated with loss of functionality, especially with a diminished proliferative capacity and loss of telomeres (56). Although the loss of the co-receptors CD27 and CD28 can be considered to be indicative of impaired telomere function in T cells and denotes progression towards replicative senescence (57), Colonna-Romano etal. (58) have reported an increase in the percentage of CD27+ B cells and NK cells during aging. Thus, since CD28 expression in our study trend to be lower in the obese groups, we speculate that chronic inflammation associated with obesity increases successive rounds of T cell proliferation and may induce exhaustion in memory CD8+ T cells, since these cells tend to lose CD28 expression before losing CD27 (57). Additionally, diet-induced obesity does not appear to affect the maintenance of pre-existing long-term memory CD8 + CD27 + T cells, as well as their cytokine production and functions (59). In this regard, since our results point to a negative correlation between the values of VO_2max_ and CD27 expression, it is possible to infer that the persistence of CD27 + cells have some involvement in the context of dysregulation of the respiratory parameter in a systemic inflammatory process as observed in obesity.

Another interesting result found in our study was the higher expression of CCR7 in the obese groups when compared to the lean subject. CCR7 is expressed mainly in including naïve T cells, central memory T cells, regulatory T cells, naïve B cells, semi-mature/mature DCs and NK cells, and a minority of tumor cells, and it acts as a key regulator guiding homeostatic lymphocytes to secondary lymphoid organs (60). Studies have shown that CCR7 expression is elevated in response to hypoxia conditions (60) and different types of cancer (61, 62). Through the use of animal models, it was possible to demonstrate the involvement of CCR7 in the process of chronic inflammation and insulin resistance. Obese mice knocked out for CCR7 -/- show a decrease in the accumulation of immune cells in adipose tissue, and consequently reduced inflammation. This observation, in turn, is related to increase in glucose uptake in these animals; suggesting that cell migration of CCR7 + populations should influence the local inflammatory process (63).

It must be taken into consideration that this study is not without limitations. First, the lack of specificity of the immunosenescence markers since we did not evaluate the immunophenotyping of circulating lymphocyte subsets. Furthermore, the lack of assessments of protein levels for the soluble mediators coded by the investigated genes and their possible correlations between RNA expression in PBMC and serum/plasma protein levels could bring a better understanding of the effects of obesity to associated early immunosenescence. However, other studies have already demonstrated that the upregulation of gene expression levels of most adipokines in PBMC is associated with their circulating concentrations, suggesting the contribution of these type of cells to the increased circulating levels in obesity states (64–66).

In conclusion, obesity, regardless of associated disease, induces increased gene expression of markers associated with inflammation and immunosenescence in circulating leukocytes in obese middle-aged individuals compared to a eutrophic group of the same age. Additionally, the increase in adipose tissue and consequently in chronic inflammation and early senescence of the immune system can lead to impairments in the cardiorespiratory capacity of obese middle-aged individuals.

## Data Availability Statement

The datasets presented in this study can be found in online repositories. The names of the repository/repositories and accession number(s) can be found in the article/supplementary material.

## Ethics Statement

The studies involving human participants were reviewed and approved by Research Ethics Committee of the University of Campinas (CAAE: 55952516.6.0000.5404). The patients/participants provided their written informed consent to participate in this study.

## Author Contributions

DB, IB, RD, KM, and CC contributed to conception and design of the study. DB and VB performed the gene expression analysis. DB and LC organized the database. DB and RD performed the statistical analysis. DB wrote the first draft of the manuscript. DB, VB, and AF wrote sections of the manuscript. All authors contributed to manuscript revision, read, and approved the submitted version.

## Funding

The authors would like to acknowledge the financial support (grant n° 2016/08751-3) and post-doctoral fellowship (grant n° 2017/06643-1) provided by the São Paulo Research Foundation (FAPESP). Additionally, the authors thank the National Council for Scientific and Technological Development (CNPq; grant n° 303571/2018-7) and the Coordination for the Improvement of Higher Education Personnel (CAPES; grant n° 102164/2020-7).

## Conflict of Interest

The authors declare that the research was conducted in the absence of any commercial or financial relationships that could be construed as a potential conflict of interest.

## Publisher’s Note

All claims expressed in this article are solely those of the authors and do not necessarily represent those of their affiliated organizations, or those of the publisher, the editors and the reviewers. Any product that may be evaluated in this article, or claim that may be made by its manufacturer, is not guaranteed or endorsed by the publisher.

## References

[1] Ng M Fleming T Robinson M Thomson B Graetz N Margono C. Global, Regional, and National Prevalence of Overweight and Obesity in Children and Adults During 1980–2013: A Systematic Analysis for the Global Burden of Disease. Lancet (2014) 384:9945. doi: 10.1016/S0140-6736(14)60460-8 PMC4624264 24880830

[2] Endalifer ML Diress G. Epidemiology, Predisposing Factors, Biomarkers, and Prevention Mechanism of Obesity: A Systematic Review. J Obes (2020) 2020:6134362. doi: 10.1155/2020/6134362 32566274 PMC7281819

[3] Rosengren A. Obesity and Cardiovascular Health: The Size of the Problem. Eur Heart J (2021) 42:34. doi: 10.1093/eurheartj/ehab518 PMC8423455 34379742

[4] Martín-Cordero L García JJ Hinchado MD Ortega E. The Interleukin-6 and Noradrenaline Mediated Inflammation-Stress Feedback Mechanism Is Dysregulated in Metabolic Syndrome: Effect of Exercise. Cardiovasc Diabetol (2011) 10:42. doi: 10.1186/1475-2840-10-42 21599899 PMC3118326

[5] Hunsche C Hernandez O de la Fuente M. Impaired Immune Response in Old Mice Suffering From Obesity and Premature Immunosenescence in Adulthood. J Gerontol A Biol Sci Med Sci (2016) 71:8. doi: 10.1093/gerona/glv082 26219848

[6] Zatterale F Longo M Naderi J Raciti GA Desiderio A Miele C. Chronic Adipose Tissue Inflammation Linking Obesity to Insulin Resistance and Type 2 Diabetes. Front Physiol (2020) 10:1607. doi: 10.3389/fphys.2019.01607 32063863 PMC7000657

[7] Gleeson M Bishop NC Stensel DJ Lindley MR Mastana SS Nimmo MA. The Anti-Inflammatory Effects of Exercise: Mechanisms and Implications for the Prevention and Treatment of Disease. Nat Rev Immunol (2011) 11:9. doi: 10.1038/nri3041 21818123

[8] Ghigliotti G Barisione C Garibaldi S Fabbi P Brunelli C Spallarossa P. Adipose Tissue Imune Response: Novel Triggers and Consequences for Chronic Inflammatory Conditions. Inflammation (2014) 37:4. doi: 10.1007/s10753-014-9914-1 PMC4077305 24823865

[9] Giordano A Murano I Mondini E Perugini J Smorlesi A Severi I. Obese Adipocytes Show Ultrastructural Features of Stressed Cells and Die of Pyroptosis. J Lipid Res (2013) 54:9. doi: 10.1194/jlr.M038638 PMC3735940 23836106

[10] Vida C González EM de la Fuente M. Increase of Oxidation and Inflammation in Nervous and Immune Systems With Aging and Anxiety. Curr Pharm Des (2014) 20:29. doi: 10.2174/1381612820666140130201734 24588831

[11] Fulop T Larbi A Dupuis G Le Page A Frost EH Cohen AA. Immunosenescence and Inflamm-Aging As Two Sides of the Same Coin: Friends or Foes? Front Immunol (2018) 8:1960. doi: 10.3389/fimmu.2017.01960 29375577 PMC5767595

[12] De La Fuente M Castro NM. Obesity as a Model of Premature Immunosenescence. Curr Immunol Rev (2012) 8:1. doi: 10.2174/157339512798991290

[13] Frasca D Ferracci F Diaz A Romero M Lechner S Blomberg BB. Obesity Decreases B Cell Responses in Young and Elderly Individuals. Obes (Silver Spring) (2016) 24:3. doi: 10.1002/oby.21383 PMC4769695 26857091

[14] Burton D Faragher R. Obesity and Type-2 Diabetes as Inducers of Premature Cellular Senescence and Ageing. Biogerontology (2018) 19:6. doi: 10.1007/s10522-018-9763-7 PMC6223730 30054761

[15] Shirakawa K Sano M. T Cell Immunosenescence in Aging, Obesity, and Cardiovascular Disease. Cells (2021) 10:9. doi: 10.3390/cells10092435 PMC8464832 34572084

[16] Arranz L Lord JM de la Fuente M. Preserved Ex Vivo Inflammatory Status and Cytokine Responses in Naturally Long-Lived Mice. Age (Dordr) (2010) 32:4. doi: 10.1007/s11357-010-9151-y PMC2980596 20508994

[17] Hazeldine J Lord JM. The Impact of Ageing on Natural Killer Cell Function and Potential Consequences for Health in Older Adults. Ageing Res Rev (2013) 12:4. doi: 10.1016/j.arr.2013.04.003 PMC4147963 23660515

[18] Rodrigues LP Teixeira VR Alencar-Silva T Simonassi-Paiva B Pereira RW Pogue R. Hallmarks of Aging and Immunosenescence: Connecting the Dots. Cytokine Growth Factor Rev (2021) 59:9. doi: 10.1016/j.cytogfr.2021.01.006 33551332

[19] Spielmann G Johnston CA O´Connor DP Foreyt JP Simpson RJ. Excess Body Mass Is Associated With T Cell Differentiation Indicative of Immune Ageing in Children. Clin Exp Immunol (2014) 176:2. doi: 10.1111/cei.12267 PMC3992037 24401077

[20] Wikby A Maxson P Olsson J Johansson B Ferguson FG. Changes in CD8 and CD4 Lymphocyte Subsets, T Cell Proliferation Responses and Non-Survival in the Very Old: The Swedish Longitudinal OCTO-Immune Study. Mech Ageing Dev (1998) 102:2–3. doi: 10.1016/s0047-6374(97)00151-6 9720651

[21] Sales-Peres SHC Azevedo-Silva LJ Bonato RCS Sales-Peres MC Pinto ACS Junior JFS. Coronavirus (SARS-CoV-2) and the Risk of Obesity for Critically Illness and ICU Admitted: Meta-Analysis of the Epidemiological Evidence. Obes Res Clin Pract (2020) 14:5. doi: 10.1016/j.orcp.2020.07.007 PMC7396969 32773297

[22] Karjala Z Neal D Rohrer J. Association Between HSV1 Seropositivity and Obesity: Data From the National Health and Nutritional Examination Surve –2008. PloS One (2011) 6:5. doi: 10.1371/journal.pone.0019092 PMC3092767 21589933

[23] Sheridan PA Paich HA Handy J Karlsson EA Hudgens MG Sammon AB. Obesity Is Associated With Impaired Immune Response to Influenza Vaccination in Humans. Int J Obes (Lond) (2012) 36:8. doi: 10.1038/ijo.2011.208 PMC3270113 22024641

[24] Huttunem R Syrjänem J. Obesity and the Risk and Outcome of Infection. Int J Obes (Lond) (2013) 37:333–40. doi: 10.1038/ijo.2012.62 22546772

[25] Palmer AK Tchkonia T LeBrasseur NK Chini EN Xu M Kirkland JL. Cellular Senescence in Type 2 Diabetes: A Therapeutic Opportunity. Diabetes (2015) 64:7. doi: 10.2337/db14-1820 PMC4477358 26106186

[26] Lau EYM Carroll EC Callender LA Hood GA Berryman V Pattrick M. Type 2 Diabetes Is Associated With the Accumulation of Senescent T Cells. Clin Exp Immunol (2019) 197:2. doi: 10.1111/cei.13344 PMC6642873 31251396

[27] Florindo AF Latorre MRDO. Validação do Questionário De Baecke De Avaliação Da Atividade Física Habitual Em Homens Adultos. Rev Bras Med Esporte (2003) 9: 121–8.

[28] Brown LE Joseph P Weir JP. Procedures Recommendation I: Accurate Assessment of Muscular Strength and Power. JEPonline (2001) 4:3. Available at: https://www.asep.org/asep/asep/Brown2.pdf.

[29] Wasserman K Whipp BJ Koyal SN Beaver WL. Anaerobic Threshold and Respiratory Gas Exchange During Exercise. J Appl Physiol (1973) 35:2. doi: 10.1152/jappl.1973.35.2.236 4723033

[30] Pradella F Boldrini VO Marques AM Moraes GAD Francelin C Cocenza RS. Cytotoxic Activity of CD4 T Cells During the Early Stage of Autoimmune Neuroinflammation. BioRxiv (2020). doi: 10.1101/2020.03.10.985614

[31] Salvestrini V Sell C Lorenzini A. Obesity May Accelerate the Aging Process. Front Endocrinol (2019) 10:266. doi: 10.3389/fendo.2019.00266 PMC6509231 31130916

[32] Reidy K Kang HM Hostetter T Susztak K. Molecular Mechanisms of Diabetic Kidney Disease. J Clin Invest (2014) 124:6. doi: 10.1172/JCI72271 24892707 PMC4089448

[33] Ronan L Alexander-Bloch AF Wagstyl K Farooqi S Brayne C Tyler LK. Obesity Associated With Increased Brain Age From Midlife. Neurobiol Aging (2016) 47:63–70. doi: 10.1016/j.neurobiolaging.2016.07.010 27562529 PMC5082766

[34] Bandt JP Monin C. Obesity, Nutrients and the Immune System in the Era of COVID-19. Nutrients (2021) 13:2. doi: 10.3390/nu13020610 PMC7917599 33668493

[35] Ventura MT Casciaro M Gangemi S Buquicchio R. Immunosenescence in Aging: Between Immune Cells Depletion and Cytokines Up-Regulation. Clin Mol Allergy (2017) 15:21. doi: 10.1186/s12948-017-0077-0 29259496 PMC5731094

[36] Maurya R Bhattacharya P Dey R Nakhasi HL. Leptin Functions in Infectious Diseases. Front Immunol (2018) 9:2741. doi: 10.3389/fimmu.2018.02741 30534129 PMC6275238

[37] Junttila IS. Tuning the Cytokine Responses: An Update on Interleukin (IL)-4 and IL-13 Receptor Complexes. Front Immunol (2018) 9:888. doi: 10.3389/fimmu.2018.00888 29930549 PMC6001902

[38] Heeb LEM Egholm C Impellizzieri D Ridder F Boyman O. Regulation of Neutrophils in Type 2 Immune Responses. Curr Opin Immunol (2018) 54:115–22. doi: 10.1016/j.coi.2018.06.009 30015087

[39] Egholm C Heeb LEM Impellizzieri D Boyman O. The Regulatory Effects of Interleukin-4 Receptor Signaling on Neutrophils in Type 2 Immune Responses. Front Immunol (2019) 10:2507. doi: 10.3389/fimmu.2019.02507 31708926 PMC6821784

[40] Heeb LEM Egholm C Boyman O. Evolution and Function of Interleukin-4 Receptor Signaling in Adaptive Immunity and Neutrophils. Genes Immun (2020) 21:143–9. doi: 10.1038/s41435-020-0095-7 PMC7274943 32139893

[41] Curfs JH Meis JF Hoogkamp-Korstanje JA. A Primer on Cytokines: Sources, Receptors, Effects, and Inducers. Clin Microbiol Rev (1997) 10:4. doi: 10.1128/CMR.10.4.742 PMC172943 9336671

[42] Williams A Greene N Kimbro K. Increased Circulating Cytokine Levels in African American Women With Obesity and Elevated HbA1c. Cytokine (2020) 128:154989. doi: 10.1016/j.cyto.2020.154989 32004791 PMC7058975

[43] Akbar AN Henson SM. Are Senescence and Exhaustion Intertwined or Unrelated Processes That Compromise Immunity? Nat Rev Immunol (2011) 11:289–95. doi: 10.1038/nri2959 21436838

[44] Wang Z Aguilar EG Luna JI Dunai C Khuat LT Le CT. Paradoxical Effects of Obesity on T Cell Function During Tumor Progression and PD-1 Checkpoint Blockade. Nat Med (2019) 25:141–51. doi: 10.1038/s41591-018-0221-5 PMC6324991 30420753

[45] Zindy F Quelle DE Roussel MF Sherr CJ. Expression of the P16^ink4a^ Tumor Suppressor Versus Other INK4 Family Members During Mouse Development and Aging. Oncogene (1997) 15:2. doi: 10.1038/sj.onc.1201178 9244355

[46] Ressler S Bartkova J Niederegger H Bartek J Scharffetter-Kochanek K Jansen-Durr P. P16ink4a Is a Robust *In Vivo* Biomarker of Cellular Aging in Human Skin. Aging Cell (2006) 5:5. doi: 10.1111/j.1474-9726.2006.00231.x 16911562

[47] Hara E Smith R Parry D Tahara H Stone S Peters G. Regulation of P16cdkn2 Expression and Its Implications for Cell Immortalization and Senescence. Mol Cell Biol (1996) 16:3. doi: 10.1128/mcb.16.3.859 PMC231066 8622687

[48] Zhu D Xu G Ghandhi S Hubbard K. Modulation of the Expression of P16ink4a and P14arf by hnRNP A1 and A2 RNA Binding Proteins: Implications for Cellular Senescence. J Cell Physiol (2002) 193:1. doi: 10.1002/jcp.10147 12209876

[49] Wu J Xue L Weng M Sun Y Zhang Z Wang W. Sp1 is Essential for P16^ink4a^ Expression in Human Diploid Fibroblasts During Senescence. PloS One (2007) 2:1. doi: 10.1371/journal.pone.0000164 PMC1764714 17225865

[50] Ksiazek K Piwocka K Brzezinska A Sikora E Zabel M Breborowicz A. Early Loss of Proliferative Potential of Human Peritoneal Mesothelial Cells in Culture: The Role of P16ink4-mediated Premature Senescence. J Appl Physiol (2006) 100:3. doi: 10.1152/japplphysiol.01086.2005 16254068

[51] Quereda V Martinalbo J Dubus P Carnero A Malumbres M. Genetic Cooperation Between p21Cip1 and INK4 Inhibitors in Cellular Senescence and Tumor Suppression. Oncogene (2007) 26:55. doi: 10.1038/sj.onc.1210578 17599058

[52] Canepa ET Scassa ME Ceruti JM Marazita MC Carcagno AL Sirkin PF. INK4 Proteins, a Family of Mammalian CDK Inhibitors With Novel Biological Functions. IUBMB Life (2007) 59:7. doi: 10.1080/15216540701488358 17654117

[53] Fordyce C Fessenden T Pickering C Jung J Singla V Berman H. DNA Damage Drives an Activin a-Dependent Induction of Cyclooxygenase-2 in Premalignant Cells and Lesions. Cancer Prev Res (Phila) (2010) 3:2. doi: 10.1158/1940-6207.CAPR-09-0229 PMC2954106 20028875

[54] Romagosa C Simonetti S López-Vicente L Mazo A Lleonart ME Castellvi J. p16^Ink4a^ Overexpression in Cancer: A Tumor Suppressor Gene Associated With Senescence and High-Grade Tumors. Oncogene (2011) 30:2087–97. doi: 10.1038/onc.2010.614 21297668

[55] Gustafson MP DiCostanzo AC Wheatley CM Kim C Bornschlegl S Gastineau DA. A Systems Biology Approach to Investigating the Influence of Exercise and Fitness on the Composition of Leukocytes in Peripheral Blood. J ImmunoTher Cancer (2019) 5:30. doi: 10.1186/s40425-017-0231-8 PMC5394617 28428879

[56] Effros RB Walford RL. T Cell Cultures and the Hayflick Limit. Hum Immunol (1984) 9:1. doi: 10.1016/0198-8859(84)90006-5 6607244

[57] Larbi A Fulop T. From “Truly Naïve” to “Exhausted Senescent” T Cells: When Markers Predict Functionality. Cytometry A (2014) 85:1. doi: 10.1002/cyto.a.22351 24124072

[58] Colonna-Romano G Bulati M Aquino A Scialabba G Candore G Lio D. Mech. Ageing Dev (2003) 124:4. doi: 10.1016/s0047-6374(03)00013-7 12714244

[59] Khan SH Hemann EA Legge KL Norian LA Badovinac VP. Diet-Induced Obesity Does Not Impact the Generation and Maintenance of Primary Memory CD8 T Cells. J Immunol (2014) 193:12. doi: 10.4049/jimmunol.1401685 PMC4372810 25378592

[60] Li Y Qiu X Zhang S Zhang Q Wang E. Hypoxia-Induced CCR7 Expression *via* HIF-1α and HIF-2α Correlates With Migration and Invasion in Lung Cancer Cells. Cancer Biol Ther (2009) 8:4. doi: 10.4161/cbt.8.4.7332 19305150

[61] Hirao M Onai N Hiroishi K Watkins SC Matsushima K Robbins PH. CC Chemokine Receptor-7 on Dendritic Cells is Induced After Interaction With Apoptotic Tumor Cells: Critical Role in Migration From the Tumor Site to Draining Lymph Nodes. Cancer Res (2000) 60(8):2209–17. Available at: https://cancerres.aacrjournals.org/content/60/8/2209. 10786686

[62] Muller A Homey B Soto H Ge N Catron D Buchanan ME. Involvement of Chemokine Receptors in Breast Cancer Metastasis. Nature (2001) 410:6824. doi: 10.1038/35065016 11242036

[63] Hellman J Sansbury BE Holden CR Tang Y Wong B Wysoczynski M. CCR7 Maintains Nonresolving Lymph Node and Adipose Inflammation in Obesity. Diabetes (2016) 65:8. doi: 10.2337/db15-1689 PMC4955992 27207557

[64] Catalán V Gómez-Ambrosi J Ramirez B Rotellar F Pastor C Silva C. Proinflammatory Cytokines in Obesity: Impact of Type 2 Diabetes Mellitus and Gastric Bypass. Obes Surg (2007) 17:11. doi: 10.1007/s11695-008-9424-z 18219773

[65] Telle-Hansen VH Halvorsen B Dalen KT Narverud I Wesseltoft-Rao N Granlund L. Altered Expression of Genes Involved in Lipid Metabolism in Obese Subjects With Unfavourable Phenotype. Genes Nutr (2013) 8:4. doi: 10.1007/s12263-012-0329-z PMC3689894 23296345

[66] Catalán V Gómez-Ambrosi J Rodríguez A Ramírez B Valentí V Moncada R. Peripheral Mononuclear Blood Cells Contribute to the Obesity-Associated Inflammatory State Independently of Glycemic Status: Involvement of the Novel Proinflammatory Adipokines Chemerin, Chitinase-3-Like Protein 1, Lipocalin-2 and Osteopontin. Genes Nutr (2015) 10:3. doi: 10.1007/s12263-015-0460-8 PMC4395559 25869413

